# Expression profiles of genes associated with inflammatory responses and oxidative stress in lung after heat stroke

**DOI:** 10.1042/BSR20192048

**Published:** 2020-06-05

**Authors:** Zhaoyu Liu, Jitao Chen, La Hu, Ming Li, Min Liang, Jianan Chen, Hai Lin, Zicheng Zeng, Weida Yin, Zhijie Dong, Jinsheng Weng, Wenxia Yao, Gao Yi

**Affiliations:** 1 Department of Center Laboratory, The Fifth Affiliated Hospital of Guangzhou Medical University, Guangzhou 510700, China; 2 Department of Respiratory Medicine, The Fifth Affiliated Hospital of Guangzhou Medical University, Guangzhou 510700, China

**Keywords:** cell enrichment analysis, heat stroke, lung, signal pathway analysis, transcriptome analysis

## Abstract

**Background**: Heat stroke (HS) is a physically dysfunctional illness caused by hyperthermia. Lung, as the important place for gas-exchange and heat-dissipation organ, is often first to be injured. Lung injury caused by HS impairs the ventilation function of lung, which will subsequently cause damage to other tissues and organs. Nevertheless, the specific mechanism of lung injury in heat stroke is still unknown.

**Methods**: Rat lung tissues from controls or HS models were harvested. The gene expression profile was identified by high-throughput sequencing. DEGs were calculated using R and validated by qRT-PCR. Gene Ontology (GO), Kyoto Encyclopedia of Genes and Genomes (KEGG), and cell-enrichment were performed using differential expression genes (DEGs). Finally, lung histopathology was accessed by H&E staining.

**Results**: About 471 genes were identified to be DEGs, of which 257 genes were up-regulated, and 214 genes were down-regulated. The most up-regulated and down-regulated DEGs were validated by qRT-PCR, which confirmed the tendency of expression. GO, KEGG, and protein–protein interaction (PPI)-network analyses disclosed DEGs were significantly enriched in leukocyte migration, response to lipopolysaccharide, NIK/NF-kappaB signaling, response to reactive oxygen species, response to heat, and the hub genes were Tnf, Il1b, Cxcl2, Ccl2, Mmp9, Timp1, Hmox1, Serpine1, Mmp8 and Csf1, most of which were closely related to inflammagenesis and oxidative stress. Finally, cell-enrichment analysis and histopathologic analysis showed Monocytes, Megakaryotyes, and Macrophages were enriched in response to heat stress.

**Conclusions**: The present study identified key genes, signal pathways and infiltrated-cell types in lung after heat stress, which will deepen our understanding of transcriptional response to heat stress, and might provide new ideas for the treatment of HS.

## Background

Heat stroke (HS) is a physically dysfunctional illness characterized by an elevated core body temperature (>40°C), feeling hot, central nervous system abnormalities (coma, fainting, and delirium), and even multiple organ dysfunction syndrome (MODS) [1–3]. Prolonged exposure to a hot environment or vigorous physical exertion are the main cause of heat stroke. Excessive accumulation of heat in the body could lead to tissue and organ damage, in severe cases, even heat stroke. With the global warming and the increasing frequency and intensity of heat wave attacks, the incidence of heat stroke is increasing year by year worldwide [4,5].

Heat stroke is a lethal illness with high rate of morbidity and mortality, The 28-day and 2-year mortality rates of heat stroke have been reported to be 58% and 71%, respectively [6]. Furthermore, heat stroke-related deaths have been reported to increase yearly as the global warming. Actually, exposure to high ambient temperatures can lead to a high incidence rate of heat stroke. In 2003, Western Europe suffered heat wave related disasters, killing more than 70,000 people [6]. Mostly recently, in 2010, heat waves in Russia caused more than 55,000 deaths [7]. By 2050s, the number of death from heat stroke is expected to raise nearly 2.5 times the current annual deaths [8].

Lung, as the most important organ for heat dissipating and oxygen exchanging, has numerous capillary networks that surround the alveoli. In the process of heat stroke, lung is one of the first affected and injured organ as its special structure, and impaired lung functions could further induce and promote the damage of other important tissues and organs as anoxia [9]. Although numerous reports have demonstrated that heat stroke causes injury to brain, small intestine, and other organs [10], the effect of heat stroke on lung is not very clear yet.

High-throughput sequencing is an emerging methodology that can provide insight into the identification of new disease-associated genes, pathways and networks that would not have been discovered with the traditional, single-analysis methods. Recent studies using this technology have been widely applied in various biological process such as development [11], airway disease [12,13], providing to be quite useful tools for the identification of potential biomarkers and therapeutic targets. In the present study, unbiased high-throughput RNA sequencing was used to evaluate the genome-wide expression of mRNA in the rat lung tissue following heat shocking.

## Materials and methods

### Animal model of heat stroke (HS) and tissue collection

**Figure 1:**
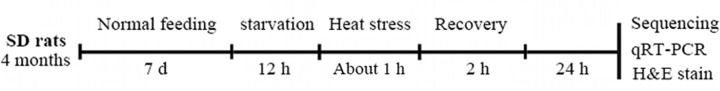
Experimental protocol Scheme of the experimental protocol for preconditioning and induction of heat stroke model. HS group rats were placed in a pre-warmed incubator that was maintained at (40 ± 0.5)°C and a relative humidity of (60 ± 5)% in the absence of food and water. When the rectal temperature was reached 42°C, rats were cooled at an temperature of (25 ± 0.5)°C and a humidity of (35 ± 5)% for temperature recovery. After 24 h, lungs were harvested for further examination.

Twenty Male Wistar rats (4 months old) were purchased from the Guangdong Medical Laboratory Animal Center (Guangzhou, China), and their weights were approximately 250 g. The animals were raised in the Center of Experimental Animals of Guangzhou medical University. The rats (5/cage) were housed under controlled environmental condition (12-h light/dark cycle, 55 ± 5% humidity, 23°C ambient temperature) with standard chow and water. All animal experiments were performed in the Center of Experimental Animals, Guangzhou Medical University, and they were approved by the Institutional Animal Care and Use Committee before the experiment. Heat stroke model were established according to previously method [14]. Briefly, all rats were starved for 12 h before experimentation, without any restriction on the ingestion of water. Twenty rats were randomly divided into two groups: control and HS. Rats in HS group were placed in a pre-warmed incubator that was maintained at (40 ± 0.5)°C and a relative humidity of (60 ± 5)% in the absence of food and water. The rectal temperature was continuously monitored at 10-min intervals with a rectal thermometer. When the temperature was more than 42°C, rats were cooled at an temperature of (25 ± 0.5)°C and a humidity of (35 ± 5)% for temperature recovery. The animals in the control group were sham-heated at a temperature of (25 ± 0.5)°C and humidity (35 ± 5)% for a time that was comparable to that of the HS groups. Twenty-four hours after model building, the rats (10-control and 10-HS rats) were anesthetized by intraperitoneal injection of pentobarbital sodium (35 mg/kg) and killed by decapitation. Then, lung tissue and blood were obtained from each rat for the subsequently experiments (Figure 1).

### RNA sequencing and data analysis

RNA extracted from lung tissue was used to construct mRNA libraries using the NEBNext Ultra II RNA Kit (Illumina platform). The RNA-seq libraries were sequenced on the Hiseq Xten platform. Raw reads were exported as fastq format, and the obtained raw reads were pre-processed by removing the adaptors and sequencing primers using Trimmomatic [15]. Low quality reads were removed to generate clean data for subsequent analysis. Reference annotation files were downloaded from genome databases website (ftp://ftp.ensembl.org/pub/release-92/fasta/rattus_norvegicus/). All of the clean reads were mapped to the Rat reference sequence using TopHat2 [16]. The read count for each gene was acquired based on the mapping results using Cufflinks and normalized to reads per kilobase of exon model per million mapped reads (RPKM) [17,18]. Differential expression genes (DEGs) between heat stroke group and control group were calculated using an R extension package ‘DESeq’ (www.bioconductor.org/packages/release/bioc/html/DESeq.html) [19], and genes with estimated absolute log2-fold change ≥ 1 and *P* value ≤ 0.05 were considered to be significantly differentially expressed. Volcano plot for all genes were drawn using R software package ‘ggplot2’, and heatmap for 50 most significant DEGs was drawn using R extension package ‘Pheatmap’.

### Enrichment of gene ontology (GO) terms and pathways

GO (Gene Ontology) analysis, including biological process (BP), cellular component (CC) and molecular function (MF), was performed for features corresponding to DEGs in heat stroke samples using the R extension package ‘clusterProfler’. The statistically significance level of GO term was set as the adjusted *P* value less than 0.05. In addition, KEGG (Kyoto Encyclopedia of Genes and Genomes) pathway analysis of DEGs was conducted to identify enriched pathways with ‘clusterProfler’ as well.

### Integration of protein–protein interaction (PPI) network

STRING (Search Tool for the Retrieval of Interacting Genes) database is an online tool designed for evaluating the protein-protein interaction information. STRING (version 11.0) includes 24,584,628 proteins from 5090 organisms. To identify the interactive relationships, DEGs were mapped to STRING, and minimum required interaction score > 0.4 was selected as significant. Subsequently, PPI networks were constructed using the Cytoscape software (Version 3.7.1). The plug-in cytoHubba was used to identify hub genes among DEGs.

### Cell types enrichment analysis

To access the heterogeneous cellular landscape in the HS lung specimens, cell types enrichment analyzes were performed. Rat gene symbols were first converted to their homologous symbols of human using an R extension package ‘biomaRt’. And then a gene signature-based method for identifying cell types from transcriptome profiles, the xCell tool (http://xcell.ucsf.edu/), was performed [20]. It performs cell type enrichment analysis for 64 immune and stroma cell types in the lung tissues. The composite score of immune cells, ImmuneScore, was obtained as well.

### Histology

Wistar rats weighing about 250 g were anesthetized intraperitoneally with sodium pentobarbital. The lung was then ventilated and in situ perfused with 0.9% NaCl until its turns white. Fresh lung was isolated and the right lung tissue was fixed for 1 day in 4% freshly prepared paraformaldehyde. Subsequently, lung tissues were dehydrated through a serial acetones gradient, embedded in paraffin wax blocks, and sections were cut at 5 μm thickness. Before Hematoxylin and Eosin (H&E) staining, lung tissue sections were dewaxed in xylene, and rehydrated through a decreasing graded series of ethanol, and rinsed in water. Then, sections were stained with H&E. After staining, sections were washed with water, and dehydrated with increasing concentration of ethanol and xylene.

### RNA extraction, reverse transcription, and quantitative PCR

Total RNA was isolated from lung tissues using Trizol reagent (Life Technologies). Reverse transcription was performed using the cDNA synthesis kit (PrimeScript™ RT reagent Kit with gDNA Eraser, TaKaRa), according to the manufacturer’s instructions. The quantitative RT-PCR (qRT-PCR) was carried out using the SYBR Premix Ex Taq (TB Green™ Fast qPCR Mix, TakaRa) and the ABI 7500 Real time PCR system as described previously [21,22]. A total of 20 genes, including ten top up-regulated and ten top down-regulated genes, were measured. GAPDH (glyceraldehyde-3-phosphate dehydrogenase) was used as an endogenous control. The relative gene expression was calculated using the 2^−ΔΔCt^ method, which represents the difference of CT (Cycle Threshold) between the control GAPDH products and the target gene products [23]. All samples were detected in triplicates, and means and standard deviations were estimated. Primers for Fbxw9 (F-box and WD repeat domain containing 9), Ccdc97 (coiled-coil domain containing 97), Pmf1 (polyamine-modulated factor 1), Tomm6 (translocase of outer mitochondrial membrane 6), Sele (selectin E), Ifnar2 (interferon alpha and beta receptor subunit 2), Fermt3 (fermitin family member 3), Nudt16I1 (nudix hydrolase 16 like 1), Ltk (leukocyte receptor tyrosine kinase), and Fbxo40 (F-box protein 40) are shown in Table 1.

**Table 1:** List of oligonucleotide primers used in the present study

Primer name	Forward (5′→3′)	Reverse(5′→3′)
Fbxw9	GACGGACAGCGGACCGAATA	AGAACTCGGCTGGGCTCCTT
Ccdc97	GACCTGCTGTTTCAGTCCTACCA	GCCCCCATCTTTGCCATCC
Pmf1	ACCATCTCCAGGGTGAAACTCC	CTCAGCCAGTTCCTGGTTCTT
Tomm6	GGCCGGTGAGAACTCCGA	GGCTGAGGTGCCATCAAATC
Sele	CTTTGACCCAACCTGCCCAC	CGATGGCTTCTCGTTGTCCC
Ifnar2	CAGCCCACCAACCAACTACAC	GCAGCGGCACACCGTTGA
Fermt3	AGCAGGCACGCTGGGATTT	CCTTGAGTTCTGGGATGGTGGT
Nudt	TCCACACCGTGTGGTGGC	TTGGCAGTGCTGACGAAGG
Ltk	CCATCAGGACAGCACCCAAC	GGAATCCCCAGGAAGACCAA
Fbxo40	TGGACAAGTTTGGCAAGTGGG	AGCCCTGTCATCTCAATGGTG

### Statistical analysis

Statistical analyses were conducted using SPSS Statistics 22 and R software (version 3.3.3). Independent sample *t* test was used for two group comparisons. All data are presented as mean ± standard deviation of at least three independent experiments. A two-sided *P* value < 0.05 was taken as statistically significant.

## Results

### mRNA expression profile in heat stroke lung tissue

**Figure 2:**
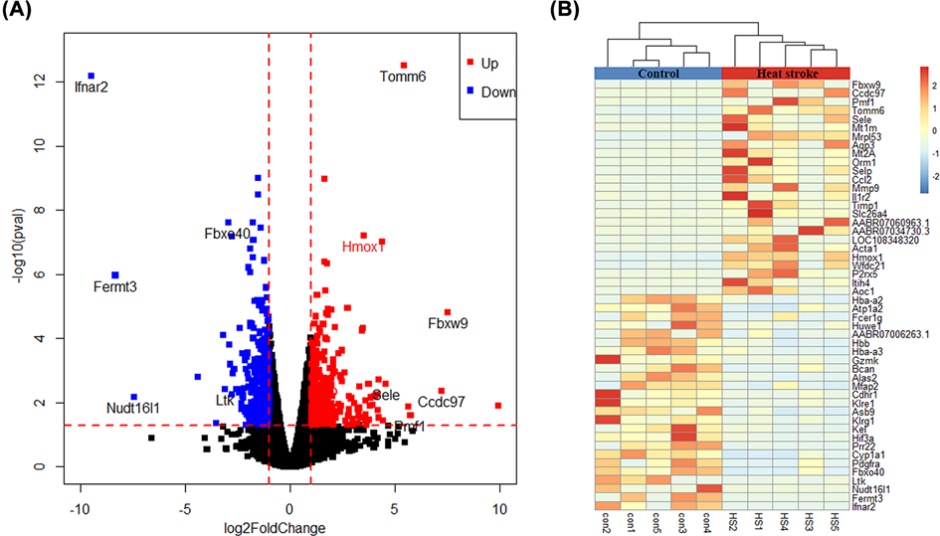
Differential expression of genes between heat stroke group and control group (**A**) Volcano diagram showing the up-regulated and down-regulated genes in heat stroke lung. The vertical axis represents the *P* value for the difference between heat stroke group and control group, and the horizontal axis represents the fold change between heat stroke group and control group. Blue points indicate genes that were down-regulated, and red points indicate genes that were up-regulated. (**B**) Unsupervised cluster analysis comparing heat stroke group and control group. Each column represents an experimental animal while each row indicates a gene. Genes with higher expression level in heat stroke group are shown in the upper part, and genes with lower expression are shown in the lower part. The color in each cell represents standardized gene expression values, red being high and blue low.

We first analyzed the profiling of mRNA in rat lung tissues by high-throughput sequencing. After the trimming of the adaptor sequences and removal of low quality reads, about 6,000,000 clean reads were acquired for each sample, of which almost 90% matched the Rattus norvegicus genome mentioned in the ‘Materials and Methods’ section. PCA analysis, Volcano plots, and heatmap showed that the mRNA expression levels were clearly distinguished and clustered between heat stroke lung specimens versus the control lung specimens. A total of 20,893 mRNAs were identified from lung specimens, of which 16,398 mRNAs were annotated before and 4495 mRNAs were previously unknown (21.5%). The annotated genes were selected for the subsequent analysis for this study. DEGs with statistical significance between the control group and heat stroke group were shown through fold change and *P* value. Based on a criteria of a fold change ≥ 2 and *P* value ≤ 0.05, a total of 471 genes were identified to be differentially expressed between control group and heat stroke group. About 257 genes were remarkably up-regulated, and 214 genes were significantly down-regulated more than 2-fold in heat stroke group compared with control specimens group. Among the most up-regulated genes were Fbxw9, CCdc97, Pmf1, Tomm6, and Sele. The most down-regulated genes were ifnar2, Fermt3, Nudt16l1, Ltk, and Fbxo40 (Figure 2A). In addition, several heat stess proteins, HMOX1 (Heme Oxygenase 1, also known as Hsp32), Hspa1a (Heat Shock 70 KDa Protein 1A) and Hp (Haptoglobin), were also identified to be differentially expressed in the present study (Figure 2A). A volcano plot of all genes is shown in Figure 2A, and a cluster heatmap of 50 most change genes is in Figure 2B, including 25 most up-regulated genes and 25 most down-regulated genes. The complete list of all DEGs and statistic symbols is available in online Supporting Information Supplementary Table S1.

### qRT-PCR validation of DEGs in heat stroke lung tissues

**Figure 3:**
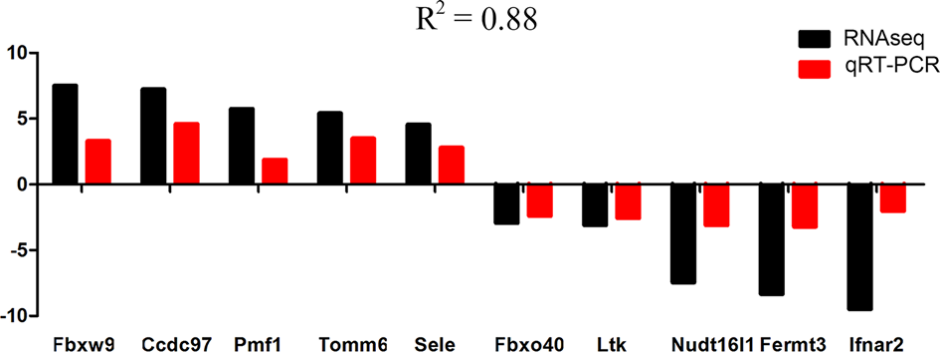
Expression levels of selected genes from RNA sequencing analysis (RNAseq, black bar) and their validation by qRT-PCR (red bar) *R*^2^ is the correlation of expression levels between log2FC value of RNA sequencing and qRT-PCR ΔCT.

Independent assays were carried out using qRT-PCR on all heat stroke specimens for ten mostly changed genes and several heat stress related genes. We validated the expression level of Fbxw9, Ccdc97, Pmf1, Tomm6, Sele, Ifnar2, Fermt3, Nudt16I1, Ltk, and Fbxo40 (Table 1). The results showed that the tendency of gene expression was consist with the RNA sequencing results, though the fold changes differed between RNA sequencing and qRT-PCR. A pearson correlation of 0.88 was acquired between log2FC value of RNA sequencing and qRT-PCR ΔCT (Figure 3).

### GO term enrichment analysis among DEGs

**Figure 4:**
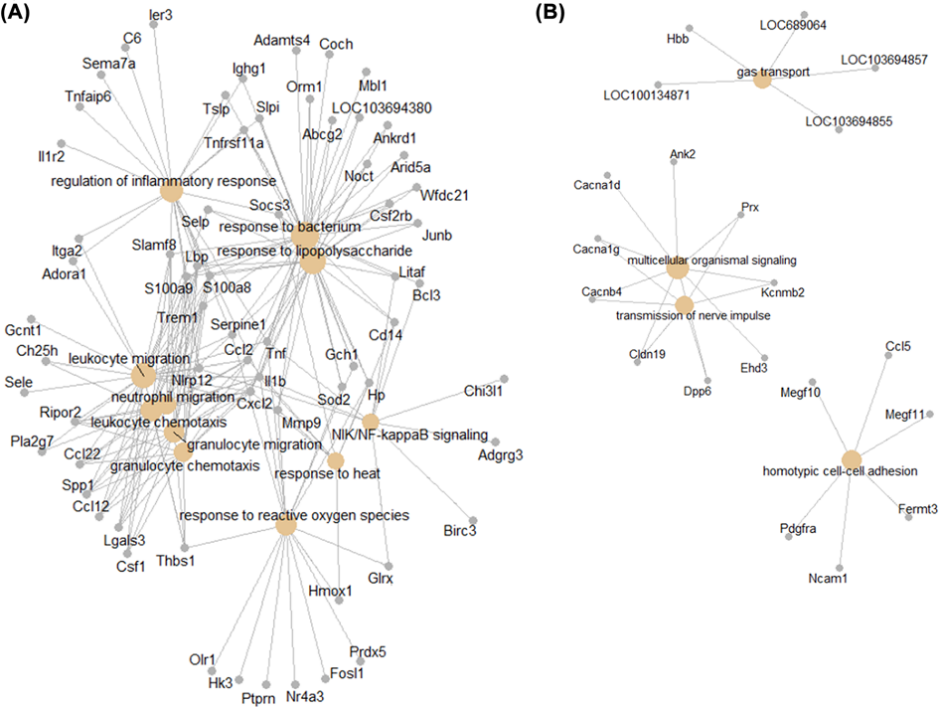
GO annotation of differential expressed genes (**A**) Up-regulated DEGs; (**B**) Down-regulated DEGs.

All DEGs were used to identify overrepresented GO categories. GO analysis results demonstrated that up-regulated genes were significantly enriched in biological processes (BP), including leukocyte migration, response to lipopolysaccharide, NIK/NF-kappaB signaling, response to reactive oxygen species and response to heat (Figure 4A and Table 2); the down-regulated genes were significantly enriched in gas transport, multicellular organismal signaling, transmission of nerve impulse and homotypic cell–cell adhesion (Figure 4B and Table 2). For molecular function (MF), the up-regulated genes were enriched in cytokine activity, receptor ligand activity and receptor regulator activity (Table 2), and the down-regulated genes were enriched in oxygen binding, carbohydrate binding and iron ion binding (Table 2). In addition, GO cell component (CC) analysis showed that the up-regulated genes were significantly enriched in extracellular matrix, and down-regulated genes were enriched in extracellular matrix, proteinaceous extracellular matrix, external side of plasma membrane (Table 2).

**Table 2:** GO enrichment analysis of DEGs in heat stroke lung tissue

Term ID	Term	nGenes	*P* value	Term name	Type
GO:0050900	BP	26	8.20E-13	Leukocyte migration	Up
GO:0032496	BP	27	2.45E-10	Response to lipopolysaccharide	Up
GO:0071621	BP	14	5.23E-10	Granulocyte chemotaxis	Up
GO:0038061	BP	10	7.98E-06	NIK/NF-kappaB signaling	Up
GO:0000302	BP	16	9.62E-06	Response to reactive oxygen species	Up
GO:0009408	BP	9	9.73E-06	Response to heat	Up
GO:0031012	CC	17	3.95E-05	Extracellular matrix	Up
GO:0005125	MF	13	1.11E-07	Cytokine activity	Up
GO:0048018	MF	18	4.30E-07	Receptor ligand activity	Up
GO:0005126	MF	13	3.97E-05	Cytokine receptor binding	Up
GO:0030414	MF	9	6.43E-05	Peptidase inhibitor activity	Up
GO:0030246	MF	13	1.11E-04	Carbohydrate binding	Up
GO:0015669	BP	5	1.82E-07	Gas transport	Down
GO:0035637	BP	9	4.73E-06	Multicellular organismal signaling	Down
GO:0019226	BP	6	6.69E-05	Transmission of nerve impulse	Down
GO:0034109	BP	6	1.37E-04	Homotypic cell–cell adhesion	Down
GO:0031012	CC	18	2.83E-07	Extracellular matrix	Down
GO:0005578	CC	14	2.60E-06	Proteinaceous extracellular matrix	Down
GO:0009897	CC	13	4.92E-05	External side of plasma membrane	Down
GO:0031225	CC	7	1.05E-04	Anchored component of membrane	Down
GO:0098552	CC	15	1.03E-03	Side of membrane	Down
GO:0019825	MF	5	3.26E-07	Oxygen binding	Down
GO:0030246	MF	14	9.82E-07	Carbohydrate binding	Down
GO:0005506	MF	9	7.04E-05	Iron ion binding	Down
GO:0004896	MF	7	9.05E-05	Cytokine receptor activity	Down
GO:0140104	MF	5	1.04E-04	Molecular carrier activity	Down

### KEGG pathway analysis among DEGs

**Figure 5:**
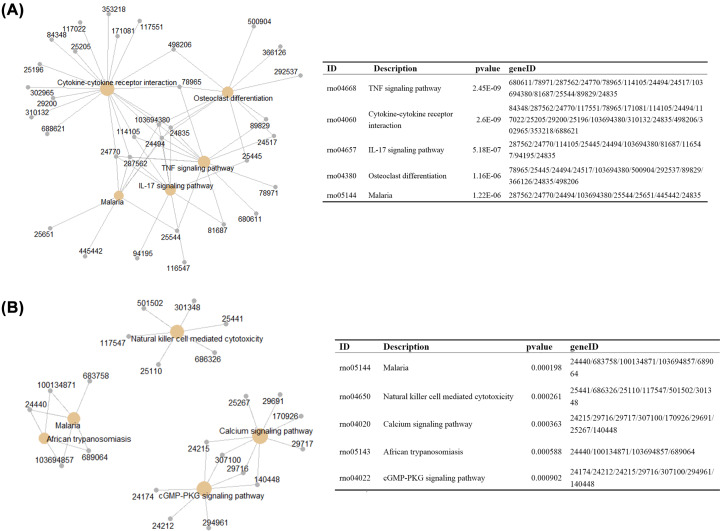
KEGG annotation of differential expressed genes (**A**) Up-regulated DEGs; (**B**) Down-regulated DEGs.

KEGG analysis was performed to identify the enriched pathways in heat stroke. The up-regulated DEGs were enriched in TNF signaling pathway, cytokine–cytokine receptor interaction, and IL-17 signaling pathway, while the down-regulated genes were enriched in Malaria, Natural killer cell mediated cytotoxicity, and Calcium signaling pathway (Figure 5 and Table 3).

**Table 3:** KEGG enrichment analysis of DEGs in heat stroke lung

ID	nGenes	*P* value	Description	Type
rno04668	13	2.45E-09	TNF signaling pathway	Up
rno04060	19	2.6E-09	Cytokine–cytokine receptor interaction	Up
rno04657	10	5.18E-07	IL-17 signaling pathway	Up
rno04380	11	1.16E-06	Osteoclast differentiation	Up
rno05144	8	1.22E-06	Malaria	Up
rno05323	8	3.35E-05	Rheumatoid arthritis	Up
rno05418	10	3.61E-05	Fluid shear stress and atherosclerosis	Up
rno04064	8	6.52E-05	NF-kappa B signaling pathway	Up
rno04145	11	6.92E-05	Phagosome	Up
rno04640	7	0.000318	Hematopoietic cell lineage	Up
rno05144	5	0.000198	Malaria	Down
rno04650	6	0.000261	Natural killer cell mediated cytotoxicity	Down
rno04020	8	0.000363	Calcium signaling pathway	Down
rno05143	4	0.000588	African trypanosomiasis	Down
rno04022	7	0.000902	cGMP–PKG signaling pathway	Down
rno04925	5	0.002064	Aldosterone synthesis and secretion	Down
rno04060	8	0.002894	Cytokine–cytokine receptor interaction	Down

### Hub genes from the PPI network

**Figure 6:**
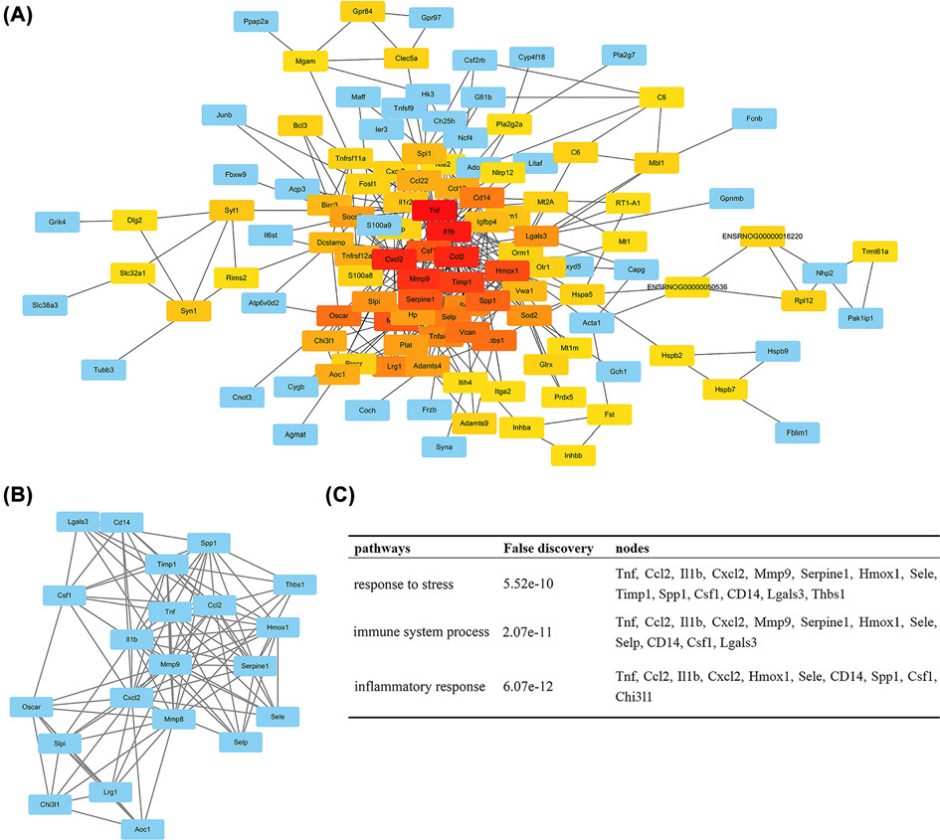
Protein–protein interaction network and a significant module (**A**) Protein–protein interaction network of differential expressed genes. Each rectangle represents a gene and the lines represent interaction relationship between genes. The color of each rectangle indicates the number of connectivity, red being high and blue low. (**B**) The most significant module selected from protein–protein interaction network. (**C**) The enriched pathway of the most significant module.

Based on the information in the STRING database, the top 10 hub nodes with higher degrees were identified. These hub nodes included Tnf (Tumor Necrosis Factor), Il1b (Interleukin 1 Beta), Cxcl2 (C-X-C Motif Chemokine Ligand 2), Ccl2 (C-C Motif Chemokine Ligand 2), Mmp9 (Matrix Metallopeptidase 9), Timp1 (TIMP Metallopeptidase Inhibitor 1), Hmox1 (Heme Oxygenase 1), Serpine1 (Serpin Family E Member 1), Mmp8 and Csf1 (Colony Stimulating Factor 1), most of which were closely related to inflammagenesis (Figure 6A). In addition, the PPI network was further analyzed using plug-ins MCODE to construct function modules. The most significant module was selected, and the functional annotation of genes involved in this module was also analyzed (Figure 6B). Enrichment analysis showed that genes were mainly associated with ‘response to stress’, ‘immune system process’, and ‘inflammatory response’ (Figure 6C).

### Differential gene expression related to inflammation and oxidative stress

**Figure 7:**
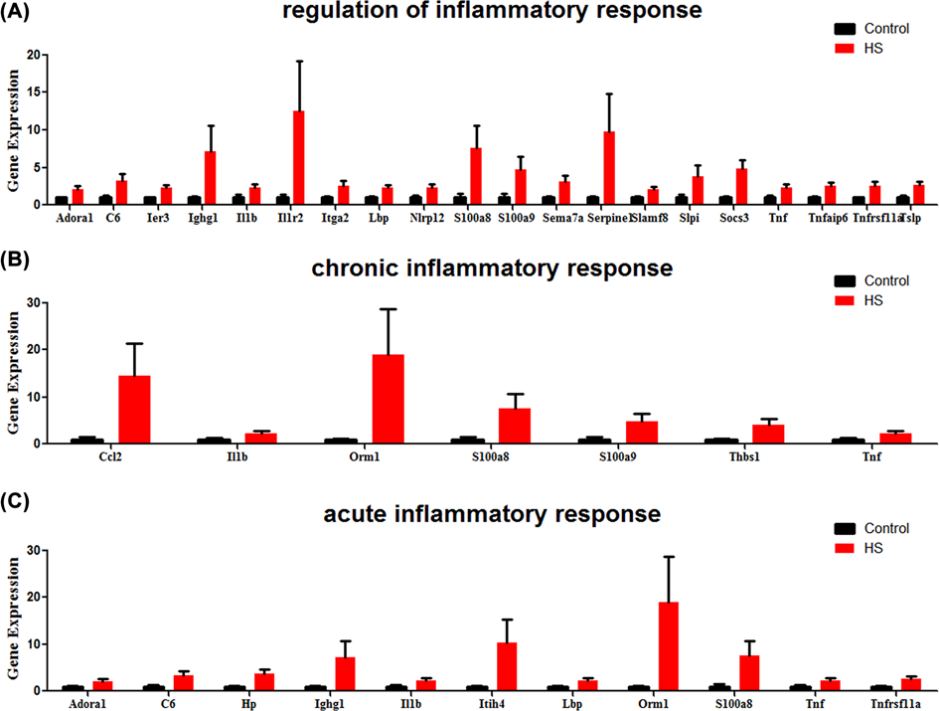
Inflammatory response genes and their relative expression in heat stroke Genes involved in (**A**) ‘regulation of inflammatory response’, (**B**) ‘chronic inflammatory response’, and (**C**) ‘acute inflammatory response’ were significantly induced in heat stroke, respectively. RNA sequencing data from heat stroke lungs and normal controls (*n* = 5 per group); data are presented in histogram with mean ± SEM.

**Figure 8:**
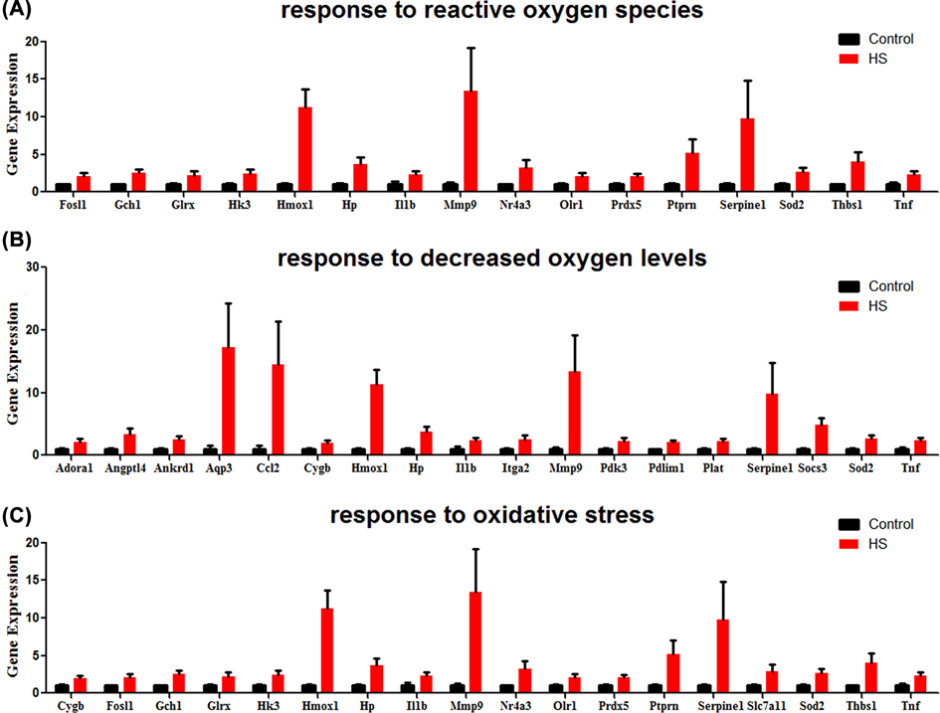
Oxidative stress genes and their relative expression in heat stroke Genes involved in (**A**) ‘response to reactive oxygen species’, (**B**) ‘response to decreased oxygen levels’, and (**C**) ‘response to oxidative stress’ were significantly induced in heat stroke, respectively. RNA sequencing data from heat stroke lungs and normal controls (*n* = 5 per group); data are presented in histogram with mean ± SEM.

Expression of genes involved in inflammation and oxidative stress were largely induced in heat stroke lung. Gene involved in ‘regulation of inflammatory response’ (Adora1, C6, Ier3, Ighg1, Il1b, Il1r2, Itga2, Lbp, Nlrp12, S100a8, S100a9, Sema7a, Serpine1, Slamf8, Slpi, Socs3, Tnf, Tnfaip6, Tnfrsf11a, Tslp), ‘chronic inflammatory response’ (Ccl2, Il1b, Orm1, S100a8, S100a9, Thbs1, Tnf), and ‘acute inflammatory response’ (Adora1, C6, Hp, Ighg1, Il1b, Itih4, Lbp, Orm1, S100a8, Tnf, Tnfrsf11a) were significantly up-regulated in heat stroke lung (Figure 7). In addition, Genes associated with oxidative stress, including ‘response to reactive oxygen species’ (Fosl1, Gch1, Glrx, Hk3, Hmox1, Hp, Il1b, Mmp9, Nr4a3, Olr1, Prdx5, Ptprn, Serpine1, Sod2, Thbs1, Tnf), ‘response to decreased oxygen levels’ (Adora1, Angptl4, Ankrd1, Aqp3, Ccl2, Cygb, Hmox1, Hp, Il1b, Itga2, Mmp9, Pdk3, Pdlim1, Plat, Serpine1, Socs3, Sod2, Tnf), and ‘response to oxidative stress’ (Cygb, Fosl1, Gch1, Glrx, Hk3, Hmox1, Hp, Il1b, Mmp9, Nr4a3, Olr1, Prdx5, Ptprn, Serpine1, Slc7a11, Sod2, Thbs1, Tnf), were also markedly induced after heat stress (Figure 8).

### Immune microenvironment in heat stroke lung

**Figure 9:**
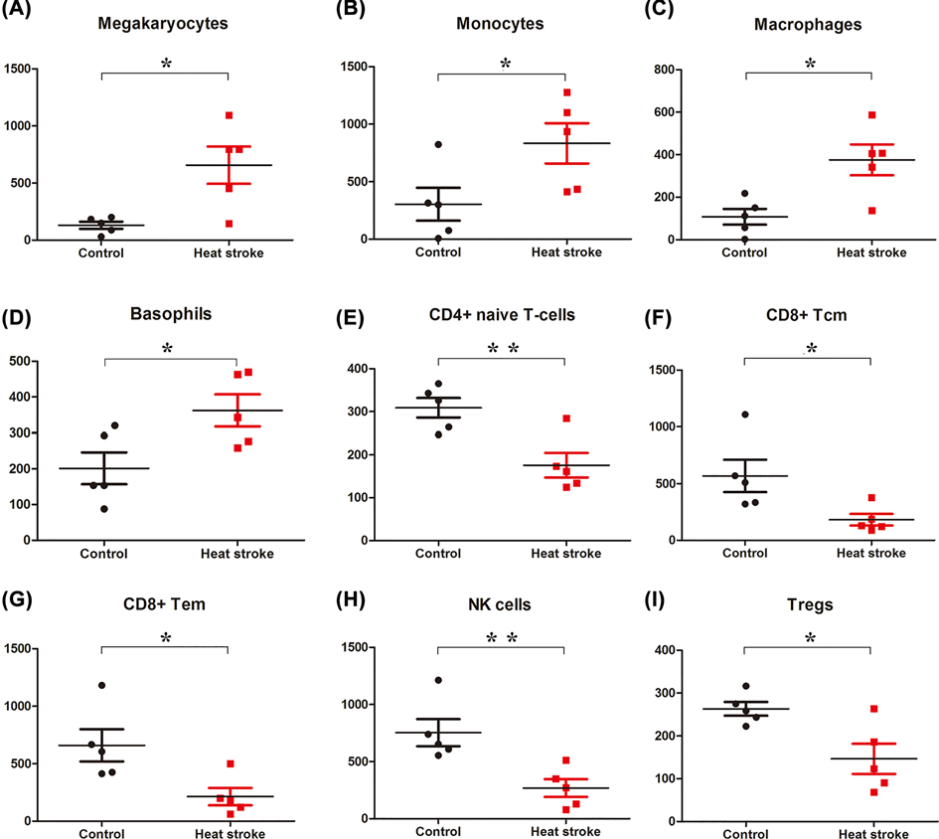
Immunity cells in heat stroke lung Immune cell fractions were detected for each specimen. Mean value and standard deviations for each cell subset including Megakaryotyes (**A**), Monocytes (**B**), Macrophages (**C**), Basophils (**D**), CD4+ naïve T-cells (**E**), CD8+ Tcm (**F**), CD8+ Tem (**G**), NK cells (**H**), and Tregs (**I**) were calculated for each group and compared using *t*-test; *P<0.05; **P<0.01.

xCell was performed to estimate of different cell types in the heat stroke lung. We found significant enrichment of Basophils, Macrophages, Megakaryocytes, and Monocytes in heat stroke lung tissue (Figure 9). Among the enriched cell types, heat stress markedly up-regulated the estimate enrichment scores of Megakaryotyes (5.0-fold, *P* value = 0.0132) (Figure 9A), Monocytes (2.7-fold, *P* value = 0.0475) (Figure 9B), Macrophages (3.5-fold, *P* value = 0.011) (Figure 9C), and Basophils (1.8-fold, *P* value = 0.0347) (Figure 9D). Inversely, CD4 T cells, CD8 T cells, and NK cells were showed a reduction trend in heat stroke lung (Figure 9), among these, the estimate enrichment scores of CD4+ naïve T-cells (0.57-fold, *P* value = 0.0066) (Figure 9E), CD8+ Tcm (0.32-fold, *P* value = 0.035) (Figure 9F), CD8+ Tem (0.32-fold, *P* value = 0.0233) (Figure 9G), NK cells (0.36-fold, *P* value = 0.0092) (Figure 9H) and Tregs (0.56-fold, *P* value = 0.0168) (Figure 9I) were reduced with statistical significance.

### Effects of heat stress on lung histopathologic changes

**Figure 10:**
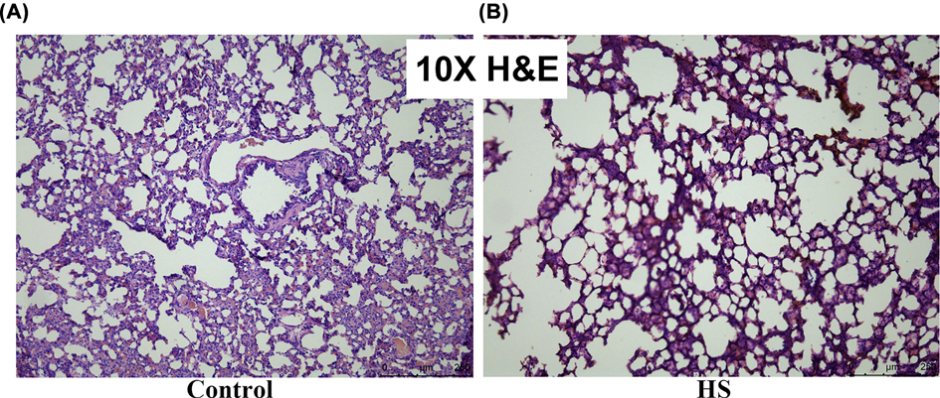
Effects of heat exposure on histopathological change in lung tissues in heat stroke Inflammation was evaluated in the lung tissues by analyzing inflammatory cell infiltration using H&E. (**A**) Control group; (**B**) Heat stroke group.

The effects of heat-mediated lung histopathologic changes were evaluated by H&E staining. As shown in Figure 10, heat stress-challenged rat showed markedly inflammatory cell infiltration and disorganized alveolar structure in lung sections. Inflammatory cells in the perialveolar, peribronchial, and perivascular were significantly elevated, and the alveolar structure was partly damaged after heat stress (Figure 10).

## Discussion

Respiration, circulation, liver, and the gastro-intestinal tract are frequently attacked and damaged by heat stroke, which are also the main process for the cause of death [1,24]. Clinical manifestations of heat stroke include acute respiratory distress syndrome (ARDS), circulation failure. Despite rapid cooling and organ support, approximately 75% of patients with severe heat stroke ultimately developed MODS [25,26]. As the important place for gas exchange, respiratory system is the most important heat dissipation organ of the body, and is most vulnerable to heat stroke [25]. Pulmonary edema and atelectasis caused by lung injury can impair the ventilation function of the lung, leading to hypoxemia and causing damage to other tissues and organs, even MODS. Therefore, a better understanding of the lung injury mechanism after heat exposure is beneficial for the early prevention and treatment of heat stroke.

In the present study, we investigated the transcriptional profiles of rat lung after heat stress, and found that 263 up-regulated DEGs and 214 down-regulated DEGs between heat stroke and normal lung tissue. These up-regulated DEGs were obviously enriched in heat stress, inflammatory response, and oxidative stress, which were intimately related to stress response. In addition, cell enrichment analysis and histopathological analysis also found several inflammatory cells, such as monocytes, macrophages and megakaryocytes, were infiltrated into lung after heat stress. Accordingly, our results demonstrated that several inflammatory pathways and inflammatory cells were enriched in rat lung after heat stress, which will provide potential diagnosis and treatment targets for heat stroke.

Pathologically, heat stroke is characterized by the dysfunction of thermal regulation, abnormal expression of heat stroke proteins, and destruction of the intestinal barrier [27]. In fact, the specific mechanism involved in heat stroke is very complicated, including a myriad of heat cytotoxicity, tissue abnormalities, and system inflammatory [10]. In consistency with other studies, our results revealed that several heat stroke proteins, including HMOX1, Hspa1a and Hp, were significantly up-regulated after heat exposure. HMOX1, which converts toxic heme to biliverdin, is a well-known gene involved in protein aggregation response under heat stress, and HOMX1 transcription refers to a lot of biological components and the activation of these components is crucial in the transcriptional regulation of HMOX1 under heat stress [28,29]. Hspa1a belongs to heat shock protein family A (Hsp70), and is important for mobilizing cytoprotection against heat stress [30]. Hp is an acute phase protein and its expression level was previously reported to be significantly elevated under heat stress in blood [31]. Meanwhile, pathway enrichment analysis also verified that pathway-related to heat response is obviously enriched.

Inflammatory injury following heat stress was the predominant pathological feature of heat stroke. Accumulating evidences have demonstrated that inflammatory damage following heat stress rather than physical burns is the main cause of organ injury in heat stroke patients and animals. Despite rapid cooling and symptomatic treatment, heat stroke always led to lethal systemic inflammatory response syndrome (SIRS), which was regarded as a response to endotoxin and bacterial infection [32]. Ameliorating pulmonary inflammation by Xuebijing, a Chinese medicinal herbs approved for use in the treatment of sepsis, could delay the development of heat stroke and increase the survival time after heat exposure [24,33]. In the present study, our findings revealed that a myriad of pro-inflammatory genes and several inflammatory-related pathways were distinctively enriched in lung under heat stress. Among these significantly up-regulated genes, 27 in 257 genes were involved in inflammagenesis. Several well-known pro-inflammatory genes, including IL1B, Tnf, CCL2 and CXCL2, were significantly up-regulated in heat exposure lung. Intriguingly, Hp, a protein related to lung defense [34], was also found to be involved in the progress of inflammation upon heat stress. We speculated that the elevated expression of Hp may reduce lung injury associated with exposure to heat stress. Analogous mechanisms have been reported to be involved in lung injury caused by pneumonia and others [35,36]. Moreover, several inflammatory-related pathways, including response to bacterium (GO:0009617), response to lipopolysaccharide (GO:0032496) and NIK/NF-kappaB signaling (GO:0038061), were also obviously enriched. Consistently, further cell enrichment analysis and histopathological analysis showed several inflammatory cells, including monocytes, macrophages and megakaryocytes, were infiltrated into lung after heat stress.

In addition to inflammatory damage, the hazard of oxidative stress in heat stroke has not been paid enough attentions. Recent studies provide that oxidative stress plays a significant role in the pathogenesis of heat stroke. Chen et al. Reported ischemic and oxidative damage to the hypothalamus is involved in the pathogenesis of heat stroke, and ischemic, hypoxic and oxidative damage to the hypothalamus might be responsible for the occurrence of MODS through hypothalamic–pituitary–adrenal axis mechanisms [37,38]. The protective effects of heat shock protein, which was significantly induced after heat exposure, was related to the attenuation of oxidative damage [39,40]. Attenuating oxidative damage by quercetin therapy improved outcomes of heat stroke in rats [41]. Our results showed a lot of oxidative stress genes (28 in 257), such as Aqp3, Cygb, SOD2, and Hmox1, were significantly up-regulated in the lung of heat exposure rat. Further study showed these genes were mainly enriched in ‘response to reactive oxygen species’, ‘response to oxidative stress’ and ‘response to decreased oxygen levels’. Aqp3 was reported to mediate hydrogen peroxide uptake to regulate down intracellular signaling [42]. Cygb was induced by hydrogen peroxide and played a protective role in oxidative stress [43]. SOD2 was induced uniquely in response to conditions of oxidative stress, underscoring the importance of protecting the energy-producing machinery within the mitochondria from oxidative damage [44]. Hmox1 was a crucial enzyme in heme catabolism that is induced by oxidative stress and had important anti-inflammatory and antioxidant effects through its metabolites in the lung [45,46]. In both *in vivo* and *in vitro* studies demonstrated that Hmox1^−/−^-deficient mice were more susceptible to oxidative injury, and exogenous delivery of Hmox1 was shown to offer protection effects upon hyperoxia-induced injury in the rat lung [47,48]. Therefore, the oxidative stress response to heat exposure appears to be an important component of the heat stroke syndromes, and can be considered in relation to the effects of heat cytoxicity.

## Conclusion

In summary, the present study provided an extensive analysis of DEGs and revealed a series of targets and pathways involved in inflammatory response and oxidative stress. This findings add to significant insights into the understanding the pathogenesis mechanism of heat stroke.

## Supplementary Material

Supplementary Table S1

## Abbreviations

DEG: differential expression genes
GO: Gene Ontology
H&E: Hematoxylin and Eosin
HS: heat stroke
KEGG: Kyoto Encyclopedia of Genes and Genomes
MODS: multiple organ dysfunction syndrome
PPI: protein–protein interaction
